# Planetary Laser Interferometric Seismoacoustic Observatory

**DOI:** 10.3390/s25010048

**Published:** 2024-12-25

**Authors:** Grigory Dolgikh, Sergey Budrin, Stanislav Dolgikh, Mikhail Bolsunovskii, Mikhail Ivanov

**Affiliations:** 1 V.I. Il’ichev Pacific Oceanological Institute FEB RAS, 690041 Vladivostok, Russia; dolgikh@poi.dvo.ru (G.D.); ss_budrin@mail.ru (S.B.); bolsunovsky.ma@poi.dvo.ru (M.B.); ivanov.mp@poi.dvo.ru (M.I.); 2 Institute of Automation and Control Processes FEB RAS, 690041 Vladivostok, Russia

**Keywords:** laser strainmeters, planetary observatory, triangulation, divergence, absorption coefficient

## Abstract

The paper describes a planetary laser interferometric seismoacoustic observatory consisting of six stationary unequal arm laser strainmeters. Based on the triangulation method, the fundamentals of direction finding of various infrasound disturbances at any planetary distance have been developed. The authors show that in addition to determining locations of the occurrence of the recorded disturbance, using data from spatially separated laser strainmeters, it is possible to determine the nature of these signals’ divergence and, also, the loss of their energy in the propagation medium. The creation of the planetary laser interferometric seismoacoustic observatory, consisting of five stationary single-coordinate laser strainmeters and one two-coordinate laser strainmeter, united into a single measuring network with an accurate time clock TRIMBLE 5700 that is capable of recording displacements on their bases with an accuracy of 10 pm in the frequency range from 0 (conventionally) to 1000 Hz and two auxiliary laser strainmeters, will allow us to determine, at any planetary distance, the primary source of deformation infrasound disturbances with primary amplitudes from 100 nm.

## 1. Introduction

Seismoacoustic oscillations of the Earth’s crust of a wide frequency range have cause-and-effect relations and serve as indicators of various physical phenomena. Such relations lie in the fact that they are both a consequence and a cause of various geophysical processes. Studying them is necessary not only for solving the inverse problem of seismoacoustics, using the parameters of oscillations and waves to study the nature of the phenomena that caused them, but also for studying the interaction of physical processes and identifying the cause-and-effect relations of emerging oscillations and waves in the formation of various geo- and biosphere phenomena on the Earth and the ambient space.

The frequency range of microoscillations of the Earth’s crust is very wide and extends from zero to hundreds of kilohertz. Each oscillation is determined by the parameters of its source, and its frequency depends on the linear dimensions of the source. Based on the amplitude-phase variations of specific oscillations, we can deduce the nature of various components’ behavior. From the point of view of the Earth’s geosphere’s physical fields interaction, a characteristic object can be taken as a singularity, possessing not only elastic properties in its classical representation but also a conglomerate of micro-objects that make up a macro-object, which in each specific case has uniform physical properties. The study of oscillation frequencies of singularities or a set of singularities is carried out by direct or indirect methods. Direct measurements are performed using inertia and inertia-free instruments and devices working on operating principles close to and far from resonance. The significant difference in the design of the instruments of these two classes is in the ability to study oscillations and waves of different frequency ranges. Inertial-type devices include various seismographs that measure oscillations and waves in a narrow frequency range. Inertia-free devices are designed to study oscillations and waves of a wider frequency range, practically from zero hertz to several kilohertz. The features of seismograph design allow amplifying the signal under study 1000 times or more, thereby increasing its ability to record oscillations and waves of small amplitude.

The era of the introduction of highly sensitive inertia-free devices can be associated with the Benioff strain seismograph [1], which was created in 1935 by Hugo Benioff at the California Institute of Technology. The working element of the Benioff meter is a fused silica cylinder, one end of which is fixed in the bedrock, and at the other end, there is a reading system that measures changes in the distance between this end and the stand, which is also installed in the bedrock.

Since the creation of the first operational, highly sensitive, inertia-free instrument for measuring microdeformations of the Earth’s crust [1], a number of mechanical strainmeters have appeared, differing from each other both in design features and in the tasks set during their design.

The peculiarity of strainmeter design implies the need to maintain stable temperature, pressure, and humidity inside the measuring room to minimize the influence of side effects on the instrument records. In addition, variations in the above parameters lead to significant changes in the dielectric constant of the air between the plates of the capacitor-sensor. The sensitivity of such an instrument is about 10^−9^ [2]. Using this strainmeter, almost the entire series of eigen oscillations of the Earth was identified after the catastrophic Chilean earthquake of 1960 [3].

An extensive international network of geophysical stations conducting continuous observations of the movements of the Earth’s crust includes several hundred rod- or wire-type strainmeters, the sensitivity of which is mainly 10^−8^ [4].

Despite the relative simplicity of their design, mechanical strainmeters have a number of serious disadvantages. These include their insufficient sensitivity in a number of applications and the very significant contribution of temperature variations to the instrument records. Thus, for the quartz strainmeter described in [2], permissible daily temperature variations, according to the description, should not exceed 0.1 K. If we take the value of thermal expansion of the strainmeter’s sensitive element material as 10^−6^, this will give, in terms of deformation, a value of 10^−7^. We can see here that the influence of temperature variations on the instrument’s readings is very strong, and careful consideration of this effect is necessary, which implies the necessity of precision control over the quartz rod temperature and recalculation of the instrument’s readings with account for errors caused by temperature instability. Considering the above, we can summarize that to ensure the commonly used sensitivity of 10^−9^, it is necessary to maintain the temperature at the location of the instrument with an accuracy of 0.001–0.0001 K.

These disadvantages, inherent in mechanical strainmeters, have necessitated the creation of a new generation of strainmeters—laser ones. The operating principle of laser strainmeters is that when the base of the strainmeter shifts, the optical path of the laser beam, traveling the distance between two points that make up the base of the device, also changes. A change in the optical path entails a change in the phase of the laser radiation wave due to an additional phase shift. This phase shift is, in most cases, the measured parameter. The primary advantage of laser strainmeters over mechanical analogs is the absence of the mechanical sensitive element. The influence of variations in meteorological parameters on the instrument’s readings comes down mainly to their impact on changes in the optical path of the laser beam. When using sealed or vacuum beam guides, the accuracy of measuring microdeformations of the Earth’s crust for the best samples is 10^−10^–10^−11^ m, with a sensitivity of 10^−12^–10^−13^ [5]. The creation of laser strainmeters of various modifications has allowed us to improve the accuracy of measuring the microdeformations of the Earth’s crust in the frequency range 0–1000 Hz by 2–3 levels of precision compared with mechanical strainmeters. The use of differential properties of the medium and specific design features of the installations gave an opportunity to increase the accuracy of measuring microdeformations of the Earth’s crust even more, while even slightly reducing the requirements for the stability of meteorological parameters (pressure, temperature, humidity), and there was a relatively low stability of the laser frequency.

The first information about the creation of a long-base laser interferometer for geophysical applications dates back to 1965 when Weili, Krogstad, and Moss [6] reported the creation of a working prototype of a laser strainmeter based on the Michelson interferometer. The operation of this device allowed for obtaining valuable data on long-term measurements of the Earth’s crust deformations [7,8,9].

Currently, several dozen variants of laser strainmeters have been created using three methods for tracking variations in the Earth’s crust deformations, with various combinations: band tracking, band counting, and frequency beat. The first measuring method was developed during the creation of the 1020 m laser strainmeter by Vali and Bostrom [8], the second was developed by King and Gerard [10], and the third was developed by Barger and Hall [11].

Along with laser strainmeters, fiber-optic measuring interferometers have become widespread [12,13]. Fiber-optic strainmeters have small dimensions and comparative protection of structures from uncontrolled external influences. The use of a fiber-optic Mach-Zender interferometer with passive phase demodulation in the sensitive element of the device makes it possible to register displacements of a section of the Earth’s crust with an accuracy of 0.5 nm in the frequency range from 0 (conditionally) to 1000 Hz [14,15]. This strainmeter allows you to register seismic signals of various origins caused by earthquakes, deformations of mountain ranges, and volcanic eruptions.

Almost all laser strainmeters currently work individually, solving narrowly defined problems. But, as some works show, the comparison of synchronous data from spatially separated laser interferometers allows for obtaining the results of planetary [16] or galactic scales [17]. Without data from spatially separated laser strainmeters, it would be impossible to obtain the unique results described in these works. Undoubtedly, the results of processing data from laser strainmeters spaced over large planetary distances will give an opportunity to obtain a number of completely new results in the geophysics, geology, and physics of the atmosphere and ocean. To perform this, it is necessary to create a system of spatially separated laser strainmeters tied into a single network. This paper describes the principle of creating such a network and its capabilities; this network consists of laser strainmeters located at the Shultz Cape station, the Primorsky Territory [18], the Svobodny station in Sakhalin area [19], near the city of Krasnokamensk [20], the Baksan area [21], and also those launched this year on Popova Island, the Primorsky Territory, and near Katsiveli settlement (Sevastopol).

## 2. Types of Laser Strainmeters

The optical design of all laser strainmeters is based on the unequal arm type Michelson interferometer and the use of a frequency-stabilized helium–neon laser.

### 2.1. Laser Strainmeter at Shultz Cape

A two-coordinate laser strainmeter is located at Shultz Cape, the Primorsky Territory, at 42°34′48.00″ N and 131°09′24.00″ E. It consists of two single-coordinate laser strainmeters of the unequal arm type with measuring arm lengths of 52.5 and 17.5 m.

The measuring arm of the 52.5 m laser strainmeter is oriented at an angle of 18° relative to the “north-south” line.

**Figure 1:**
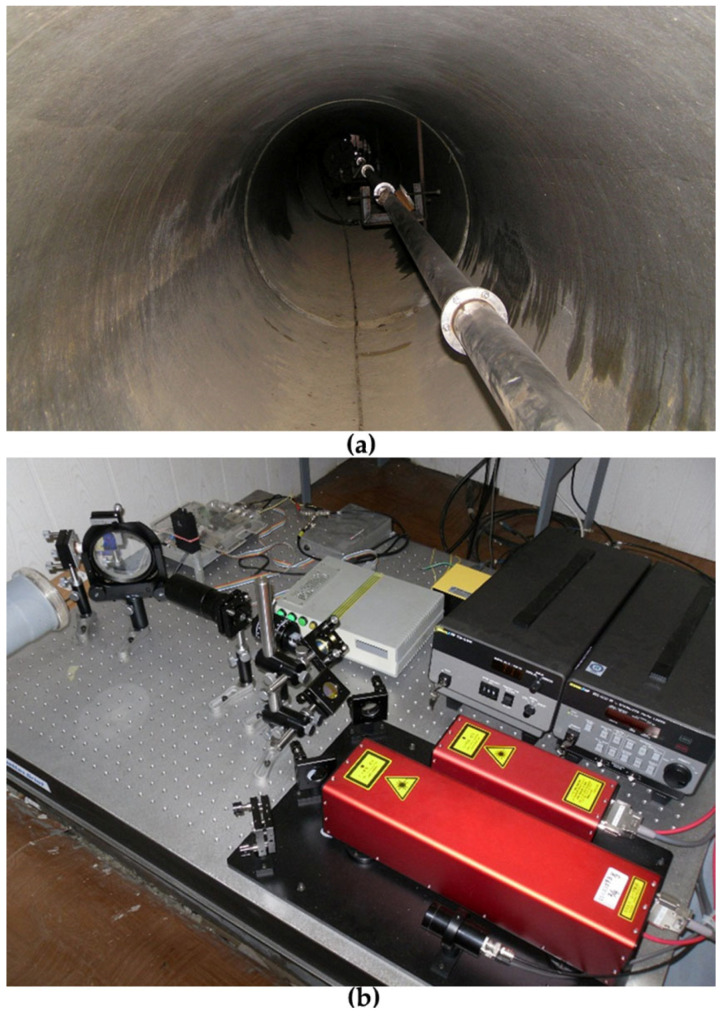
Underground beam guide of the 52.5 m laser strainmeter of the unequal arm type (**a**) and the central interference unit (**b**).

All structural elements of the laser strainmeter are located underground at a depth of 3–5 m from the surface. The corner reflector and the central interference unit of the laser interferometer are located underground in separate hydrothermally insulated rooms (see, for example, Figure 1b). The corner reflector is located on a concrete base approximately 1 m tall, which is firmly connected to the granite rock. The central interference unit is mounted on a granite–concrete abutment about 3.5 m tall. The laser beam between the central interference unit and the corner reflector propagates in the vacuum/air-filled beam guide assembled from stainless steel pipes with an internal diameter of 9 cm (see Figure 1a). Using modern interferometry methods makes it possible to measure displacement variations between the abutments of the laser strainmeter with an accuracy of 10 pm.

**Figure 2:**
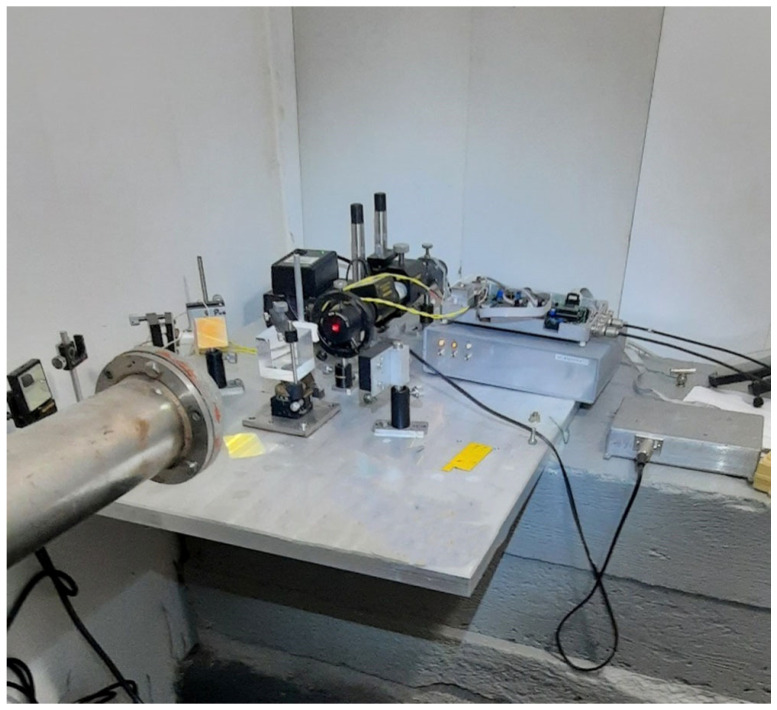
Central interference unit of the 17.5 m laser strainmeter.

The measuring arm of the 17.5 m laser strainmeter is oriented at an angle of 92° relative to the axis of the 52.5 m laser strainmeter, or 110° relative to the “north-south” line. All structural elements of the laser strainmeter are located underground at a depth of 3–5 m from the surface. The underground room of this laser strainmeter is located at a distance of about 70 m from the underground room of the 52.5 m laser strainmeter. The corner reflector and the central interference unit are mounted on granite–concrete blocks 1.5 m tall, firmly connected to the bedrock of Shultz Cape. Figure 2 shows a photograph of the central interference unit of the 17.5 m laser strainmeter. The measurement accuracy of this laser strainmeter is the same as that of a 52.5 m laser strainmeter.

### 2.2. Krasnokamensk Laser Strainmeter

**Figure 3:**
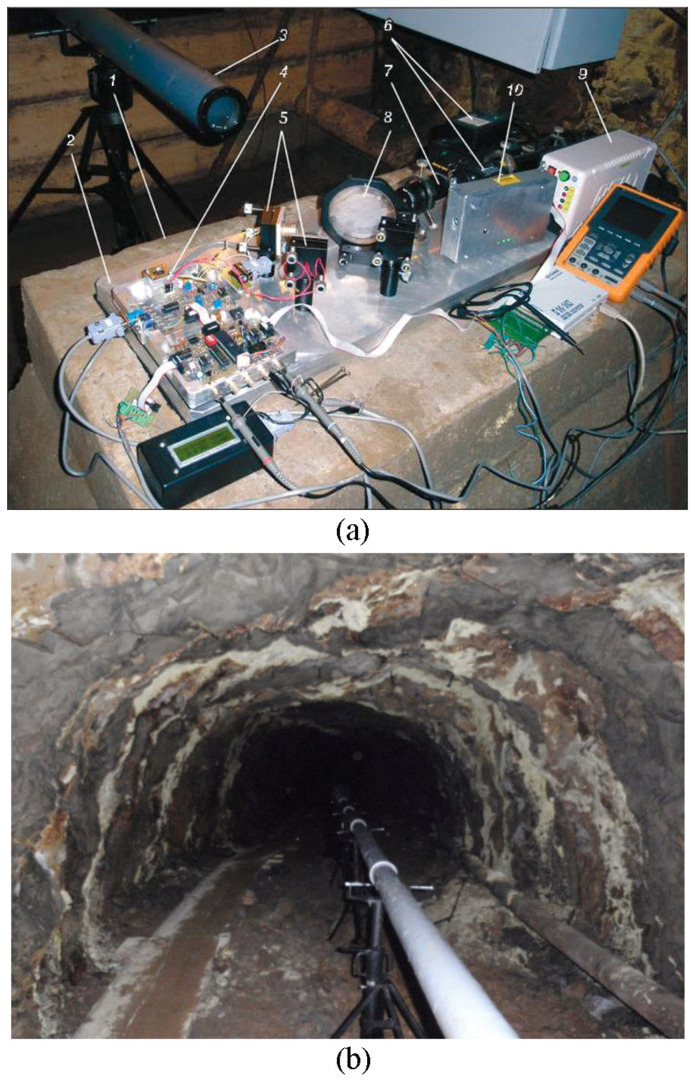
Central interference unit of the Krasnokamensk laser strainmeter (**a**) and its laser beam guide (**b**). 1—concrete foundation, 2—optical plate, 3—sealed beam guide, 4, 9, 10—registration system, 5—piezoceramics of compensation and control, 6—frequency-stabilized laser, 7—collimator, 8—PI-100 dividing plate.

In the underground mine of the PJSC Priargunsky Industrial Mining and Chemical Association (Krasnokamensk), at a depth of more than 300 m, a 50 m laser strainmeter was installed and tested, the optical part of which was assembled on the basis of a modified Michelson interferometer and a frequency-stabilized laser from MellesGriot. This laser strainmeter, capable of recording variations in the deformations of the Earth’s crust in the frequency range from 0 (conventionally) to 1000 Hz with an accuracy of 10^−10^, was a part of the complex designed to detect impending man-made disasters in the area of operating uranium mines and those under construction. In addition to their practical significance, the obtained experimental data are used to address various fundamental problems. Figure 3a shows a photograph of the central interference unit of the laser strainmeter during adjustment works, and Figure 3b shows a photograph of the laser beam guide. The laser strainmeter is located at 50°03′14.00″ N and 118°10′31.00″ E, and its measuring axis is at an angle of 30° relative to the “north-south” line.

### 2.3. Laser Strainmeter at Svobodny Cape

**Figure 4:**
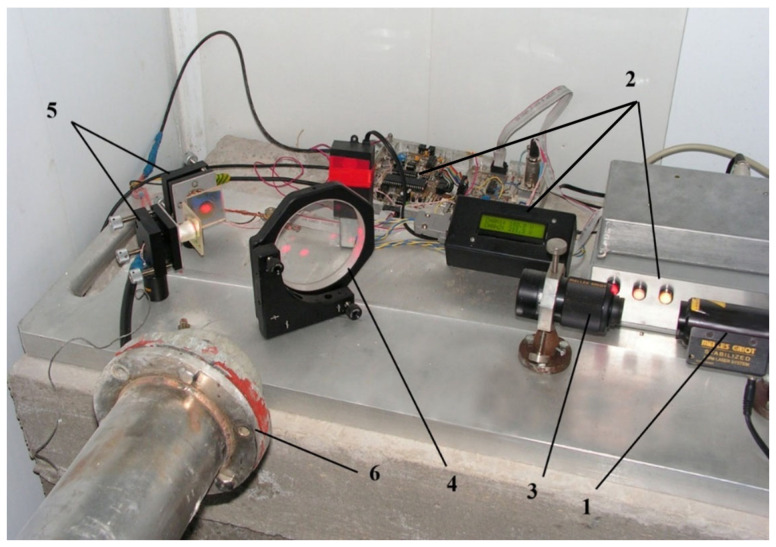
Central interference unit of the laser strainmeter. 1—frequency-stabilized laser, 2—registration system, 3—collimator, 4—PI-100 dividing plate, 5—piezoceramics of compensation and control, 6—sealed beam guide.

At Svobodny Cape on Sakhalin Island at 46°50′54.00″ N and 143°25′57.00″ E, a 20 m laser strainmeter of the unequal arm type was installed at a depth of 3 m from the ground surface in a specially built underground room. The measuring axis of the laser strainmeter is at an angle of 9° relative to the “north-south” line. Figure 4 shows a photograph of the central interference unit of the laser strainmeter. The optical design of the laser strainmeter is based on the unequal arm type Michelson interferometer using a frequency-stabilized helium–neon laser. Using modern interferometry methods makes it possible to measure displacement variations between the strainmeter abutments, on which a corner reflector and a central interference unit are installed, with the same accuracy. A detailed description of the principle of construction and operation of the device is described in the work by [19].

### 2.4. Baksan Laser Strainmeter

**Figure 5:**
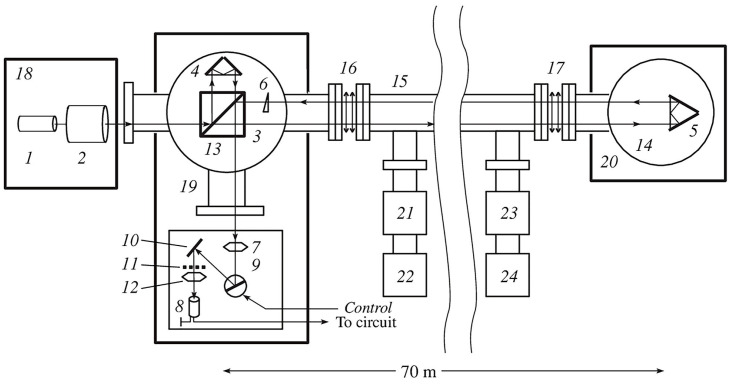
Baksan laser strainmeter. 1—He-Ne laser; 2—telescopic system; 3—beam splitter; 4, 5—corner reflectors; 6—prism; 7—lens; 8—photodiode; 9—galvanometer; 10—rotating mirror; 11—raster; 12—lens; 13, 14—vacuum chambers; 15—vacuum pipes; 16, 17—bellows elements; 18, 19, 20—concrete foundations; 21, 22, 23, 24—vacuum pumps.

Figure 5 shows the layout of the Baksan laser strainmeter. This interferometer–strainmeter with a measuring base of 75 m and a resolution of 3 × 10^−12^ is located in the underground gallery of the Baksan Neutrino Observatory (North Caucasus) near the dormant Elbrus volcano. Interferometer coordinates are 43°16′37.00″ N and 42°41′35.00″ E. The measuring arm is located at an angle of 150°37′ to the “north-south” line. A detailed description of the principle of construction and operation of the device is described in the work by [21].

### 2.5. Laser Strainmeter on Popova Island and near Katsiveli Settlement

Both laser strainmeters are currently being configured and should be launched into the mode of conducting continuous measurements of variations in the deformations of the Earth’s crust in the frequency range from 0 (conventionally) to 100 Hz and more. Both laser strainmeters have an accuracy of 10 pm in measuring displacement variations on the measuring base of the unequal arm Michels laser interferometer. The measuring arm of the laser strainmeter on Popova Island has a length of 20 m, and the laser strainmeter near Katsiveli settlement is 30 m. The operating principle of these laser strainmeters is similar to that of laser strainmeters located at Shultz Cape.

The laser strainmeter on Popova Island is located at 42°28′45.00″ N and 131°43′30.00″ E, and its measuring axis is at an angle of 46° relative to the “north-south” line.

The laser strainmeter near Katsiveli settlement is located at 44°23′24.00″ N and 33°58′48.00″ E, and its measuring axis is at an angle of 29° relative to the “north-south” line.

The registration systems of all laser strainmeters are “tied” to the exact time clock (GPS TRIMBLE 5700), providing time accuracy of the obtained data of 1 μs, which is quite sufficient for studying the phase changes of planetary signals by the laser strainmeters separated over long distances.

Table 1 summarizes the data of all laser strainmeters included in the planetary observatory.

### 2.6. Quantitative Sensitivity Analysis

**Figure 6:**
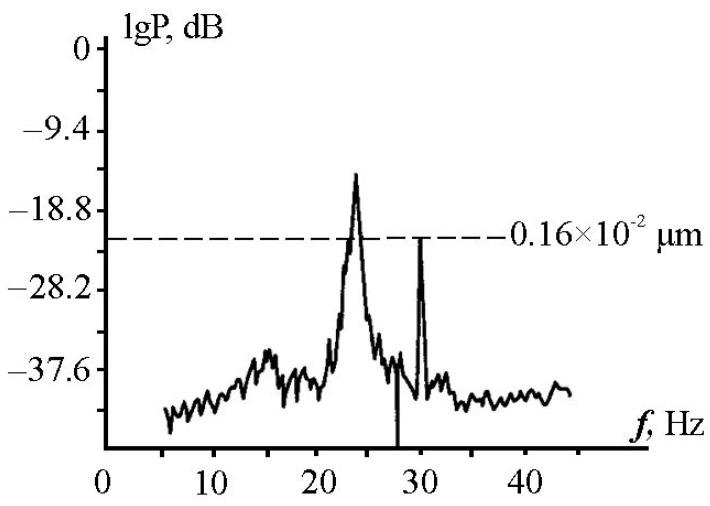
The recording spectrum of a 52.5 m laser strainmeter.

All laser interferometers, when evaluating the resulting displacement, are “tied” to the wavelength of the frequency-stabilized laser used. The 52.5 m laser strainmeter installed on cap Schultz uses a frequency-stabilized laser with long-term frequency stability in the eleventh sign and short-term stability in the twelfth sign. With such stability, it is safe to talk about isolated signals at the level of 52.5 pm, i.e., these are signals of unknown origin that are not related to the instability of the laser frequency. But, when we know about the frequency of the recorded signal, we can talk about the best measurement accuracy associated with the possibility of interference methods, which theoretically can give an accuracy of 1 pm. We can verify this statement by registering artificial signals at a given frequency. We conducted similar experiments en masse, but they were related to the study of the possibilities of recording signals emitted in water and transforming them into seismic acoustic signals at the water-bottom boundary. We are talking about sonar signals emitted by low-frequency sonar emitters at various distances from the shore at various depths of the sea. We worked with emitters that create signals of varying complexities in water (harmonic, sweep signals, phase manipulated) at frequencies of 16–25, 33–35, and 240–250 Hz. As one example of the isolated signal from the recording of a laser strainmeter at a frequency of 32 Hz, Figure 6 shows a spectrum showing a powerful peak at a frequency of 32 Hz with an amplitude of 16 nm. Taking into account the peak value of 20 dB, the base of this peak starts at 160 pm, but this is not the background level. As can be seen in Figure 6, the background level corresponds to 26 dB, i.e., it is at the level of 50 pm. Of course, this level depends not only on the accuracy of the measurement but, most importantly, on the noise level in a specific range at a specific time.

**Figure 7:**
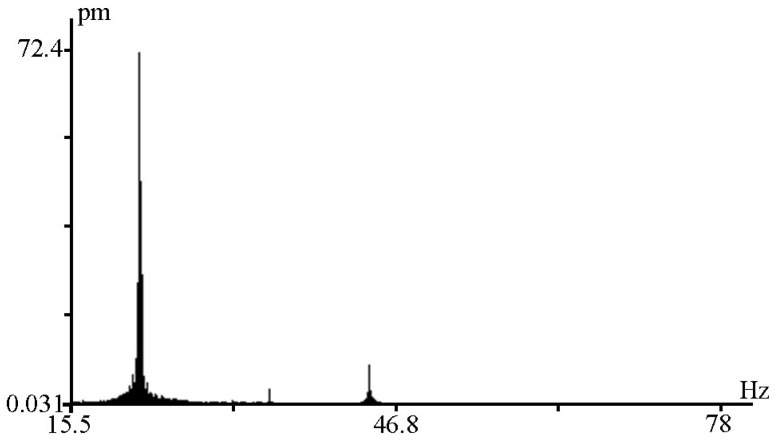
Registration of the hydroacoustic emitter signal on the recording spectrum of a laser strainmeter.

When recording signals generated in water by a hydroacoustic emitter operating at a frequency of 22 Hz, maxima with lower amplitudes were identified during spectral processing. As a typical example, Figure 7 shows a spectrum showing the first maximum at a frequency of 22.0 Hz with an amplitude of 72 pm, the second maximum at a frequency of 34.4 Hz with an amplitude of 3.2 pm, and the third maximum at a frequency of 43.9 Hz with an amplitude of 8.3 pm.

## 3. Operating Principle of Planetary Laser Interferometric Seismoacoustic Observatory: Triangulation

**Figure 8:**
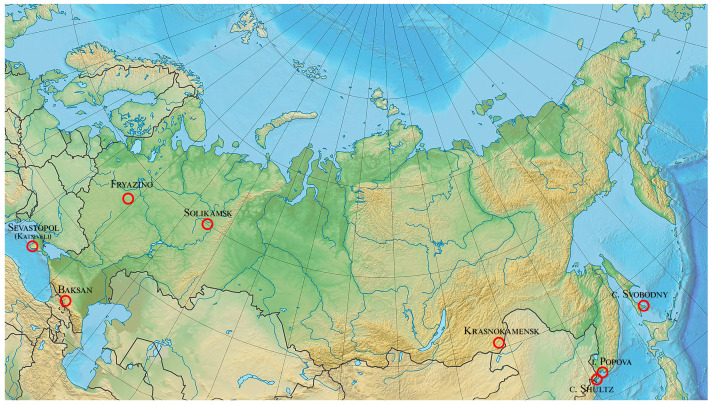
Schematic map of the laser strainmeter locations. The red circle marks the installation locations of the laser strainmeters.

Figure 8 shows a schematic map on which the locations of the above laser strainmeters are plotted. In this part of the paper, we will describe the principle of recording with these laser strainmeters any oscillations and waves propagating through the Earth’s crust in the areas of the instruments’ locations, with the capability to determine their main parameters (amplitude and frequency) and find the direction to these oscillations and waves sources.

It is clear that with the same load, the recorded displacement will depend on the length of the measuring arm of the laser strainmeter. The magnitude of the recorded displacement of the 75 m laser strainmeter will be 1.5 times greater than the recorded displacement of the 50 m laser strainmeter. This applies to all laser strainmeters, but only provided that they are all installed on rocks with exactly the same characteristics. It is clear that this condition is not met, but approximate estimates should be given when comparing signals of the same type recorded by spaced-apart laser strainmeters; we will perform it further.

The main purpose of the planetary laser interferometric seismoacoustic observatory is to determine the main characteristics of various deformation signals propagating over planetary distances, with the ability to determine the location of their origin. Below, we give an example of such calculations when recording a surface longitudinal wave with all laser strainmeters. In this case, we will assume that this wave does not change the direction of propagation on inhomogeneities of the Earth’s crust, which can be true for infrasound waves having long wavelengths. We will take into account that these waves, being surface waves, with distance are subject to cylindrical divergence. Moreover, we will introduce possible losses associated with the scattering/absorption of the energy of this wave by the medium of its propagation.

### 3.1. Triangulation Method of Finding Direction to Wave Source

Currently, almost all of the above laser strainmeters that are part of the planetary seismoacoustic observatory have one measuring arm, i.e., are single coordinates. Therefore, to find the direction of the source of oscillations/waves recorded by all laser strainmeters, only the triangulation method is applicable. We will consider the principle of its application using the example of laser strainmeters located on Sakhalin, near Krasnokamensk, and at Shultz Cape, which represents the eastern part of the planetary observatory, and of the installations of the western part of the planetary observatory (Baksan, Sevastopol). We will carry out calculations for any disturbances recorded by the considered laser strainmeters (surface waves, volume waves, pulses, etc.).

Many dangerous geo- and hydrodynamic phenomena, both of natural (earthquakes, volcanic eruptions) and anthropogenic origin (underwater and underground explosions), may be sources recorded by laser strainmeters. In this example, as a source, we will use the eruption of the active Bezymyannaya Sopka volcano, located on the Kamchatka Peninsula at 55°58′0.00″ N and 160°37′60.00″ E. We will assume that oscillations from the source penetrate into the upper layer of the Earth’s crust and propagate to the receiving points with an average speed of 3.174 km/s. We will choose three laser strainmeters as receiving systems, namely, Shultz Cape, the Primorsky Territory; Svobodny Cape, Sakhalin Island; and Krasnokamensk, the Zabaykalsky Territory.

Initially, we do not know the distance to the source; accordingly, we can only operate with the difference in the oscillation’s arrival time between laser strainmeters. To ensure the reliability of the results, we will compile a table (Table 2) where we will enter data on the distances and arrival times (*t*) from the source to each of the receivers.

**Figure 9:**
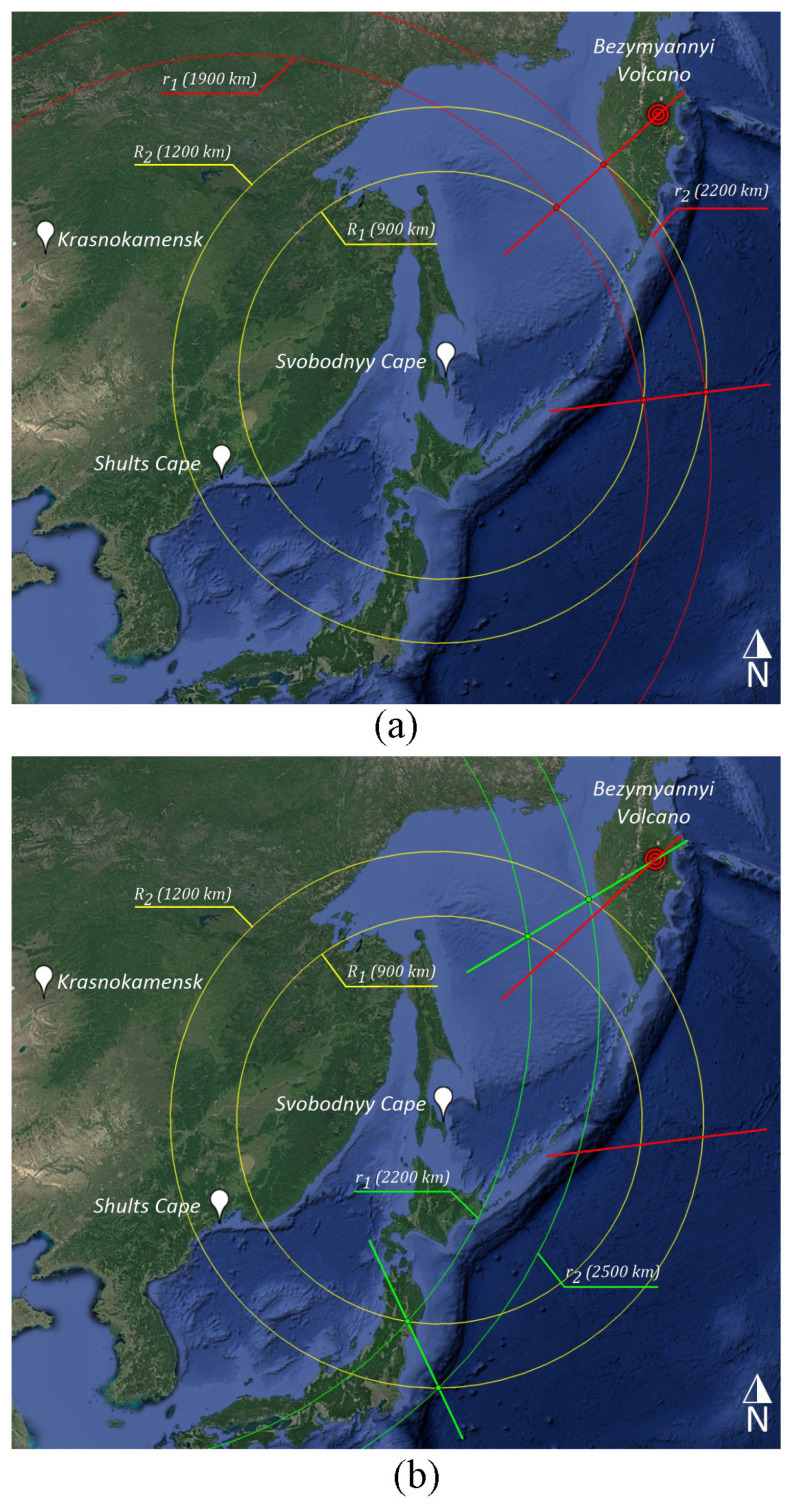
Scheme of finding directions to the source along propagation paths “Svobodny Cape-Shultz Cape” (**a**) and “Svobodny Cape–Krasnokamensk” (**b**). Yellow circles of Svobodny Cape point. Red circles Shultz Cape point. Green circles Krasnokamensk point. The red and green lines represent the direction to the source.

Based on geographical constructions (Figure 9), the disturbance will arrive first at the strainmeter located at Svobodny Cape; accordingly, we take this point as “zero”, and from it, we will calculate the distances (*S*) and arrival times (Δ*t*) to other receiving systems. Calculations of these values are also presented in Table 2.

For further constructions, we will select two arbitrary reference radii of *R*_1_ = 900 km and *R*_2_ = 1200 km, which will be used in calculations, and the time of oscillation propagation from the source to each of the strainmeters will be denoted as *t*_1_, *t*_2_, and *t*_3_, respectively. The propagation time of the disturbance along the “Svobodny Cape–Shultz Cape” and “Svobodny Cape–Krasnokamensk” paths will be denoted as Δ*t*_1_ = *t*_2_ − *t*_1_ and Δ*t*_2_ = *t*_3_ − *t*_1_ (Table 2, column 5). The distance that the oscillations have traveled along the same paths will be denoted as *S*_1_ = Δ*t*_1_ × *V* and *S*_2_ = Δ*t*_2_ × *V* (Table 2, column 6), where *V* is the speed of the propagation (3.174 km/s).

To find the first pair of directions to the source, we construct concentric circles on the schematic map (Figure 9a) with reference radii (*R*_1_, *R*_2_), with the center in the point “Svobodny Cape” and a circle with the center in the point “Shultz Cape” with radii of *r*_1_ = Δ*t*_1_ × *V* + *R*_1_ and *r*_2_ = Δ*t*_1_ × *V* + *R*_2_. The same calculations of radii but using the formulas *r*_1_ = Δ*t*_2_ × *V* + *R*_1_, and *r*_2_ *=* Δ*t*_2_ × *V* + *R*_2_ will be carried out for the point “Krasnokamensk”. Thus, we obtain two pairs of concentric circles with radii of 1900, 220 km for the point “Shultz Cape”, and radii of 2200, 2500 km for the point “Krasnokamensk”.

As we can see in Figure 9a, the reference circles (*R*_1_, *R*_2_) and the circles calculated using the above formulas (*r*_1_, *r*_2_) have two pairs of intersection points through which we draw two rays, which are the assumed directions to the source. One of these directions is false. To get rid of this uncertainty and localize the source, we perform the same calculations and constructions for the propagation path “Svobodny Cape–Krasnokamensk” (Figure 9b). As a result of this construction, we obtain the intersection of two rays of directions, which tells us that this direction is “true”, and the source of oscillations is located in the area of intersection of these segments. This area can range from several kilometers to several dozen kilometers. To increase the accuracy of determining the area in which the source is located, it is necessary to increase the number of receiving systems. As a result, the intersection of the constructed rays will form a source area, which will decrease with an increase in the number of receivers.

For this method to work correctly, it is necessary for one of the receivers to be positioned within the area bounded by the reference radii, as shown in Figure 9 (Shultz Cape); the source at the same time must be located outside this area, and it does not matter if it is in front of or behind it. If these conditions are not met, then the calculated radii (*r*_1_, *r*_2_) will coincide with the reference ones, i.e., will have no intersections with them.

From all of the above, we can conclude that the triangulation method should work well with spatially separated systems of laser strainmeters, thus allowing us to localize the sources of dangerous geo- and hydrodynamic phenomena of natural and anthropogenic nature.

Following the above, using the triangulation method, we will determine the directions to the place of generation of the signal recorded by the specified laser strainmeters. Some correction/checking of the obtained result can be performed based on the data of the two-coordinate laser strainmeter from which the direction to the source of the recorded signal was determined independently of the triangulation method. But, at each observation point, we will register the relative amplitude of this signal wave. Taking into account the direction to the source and location of the axis of each laser strainmeter relative to this direction, we can calculate the amplitude of the passing wave, accounting for the fact that we are recording a Rayleigh wave with longitudinal polarization (or a Love with transverse polarization). Having determined the amplitude of the wave at each recording point, we can evaluate the patterns of propagation of this signal from point to point, i.e., determine the nature of divergence and estimate the possible attenuation coefficient in the medium of propagation. Taking into account the fact that we are recording surface waves, we can expect that the signal will be subject to cylindrical divergence, in which the wave amplitude will decrease with distance in proportion to $1/\sqrt{R}$, where *R* is the distance in meters, and we will take into account scattering/absorption when introducing the multiplier $e^{-kR}$, where k is the absorption coefficient.

As indicated above, laser strainmeters located near Katsiveli settlement (Sevastopol) and in Baksan are also part of the planetary laser seismoacoustic observatory and represent its western part. When interpreting the obtained results, we can use data from additional laser strainmeters located at the Fryazino test site [22] and in the Urals (Solikamsk) [16]. All these laser strainmeters are of the single-coordinate type; therefore, to determine the direction of the source of the disturbances recorded by them, it is necessary to use the triangulation method. Below, we will demonstrate the possibility of its use on the specified laser strainmeters. In this case, we will consider using four laser strainmeters operating in Baksan, Sevastopol, Fryazino, and Solikamsk, and we will show how increasing the number of registration points improves the direction finding to the disturbance source.

We also assume that the propagation speed of the disturbance is 3.174 km/s, and as a source, we will take a seismic disturbance that occurred at the bottom of the Caspian Sea in a place with coordinates of 37°35′18.42″ N and 53° 3′46.80″ E. Next, we have the following:

Distance from source to receiving points.

Source—Baksan           1090 km

Source—Sevastopol     1810 km

Source—Fryazino         2330 km

We calculate the time of arrival at each point.

Source—Baksan            1090/3.174 = 343 s

Source—Sevastopol      1810/3.174 = 570 s

Source—Fryazino          2330/3.174 = 734 s

We take into account that Baksan is point “0”, i.e., subtract the time of the wave arrival to Baksan from the remaining arrival times.

Baksan             0 s

Sevastopol      570 − 343 = 227 s

Fryazino          743 − 343 = 390 s

### 3.2. Baksan–Sevastopol

**Figure 10:**
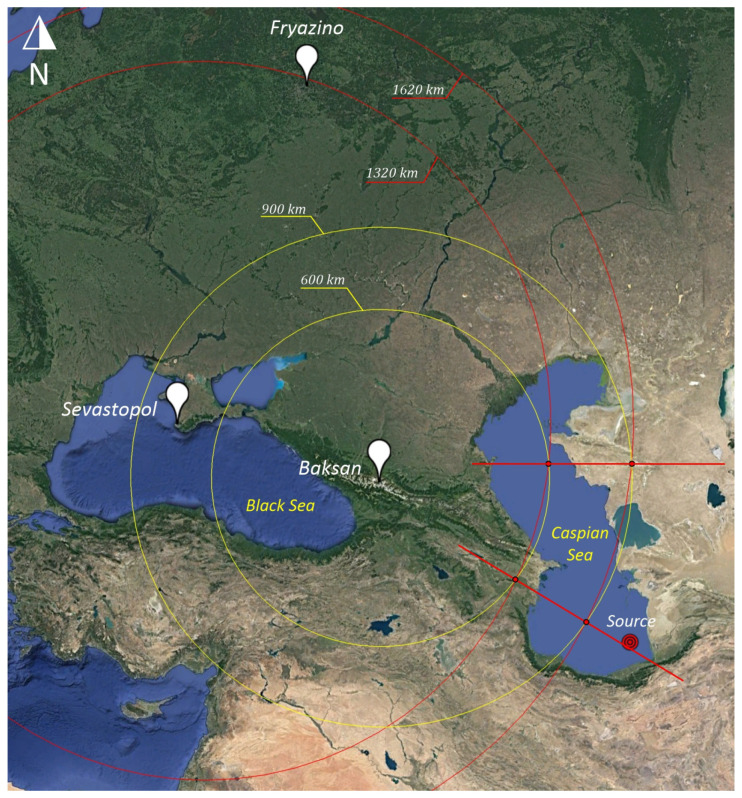
Laser strainmeters “Baksan-Sevastopol”. Yellow circles of Baksan point. Red circles Sevastopol point. The red lines represent the direction to the source.

We draw two circles with the center of the point Baksan with radii of 600 km and 900 km, as shown in Figure 10. We calculate the distance taking into account the time of propagation from Baksan to Sevastopol (227 × 3.174 = 720 km). We draw two circles with the center of the point Sevastopol with radii of 600 + 720 = 1320 km and 900 + 720 = 1620 km. By the intersections of the circles, we draw two directions to the true source (red lines).

### 3.3. Sevastopol–Fryazino

**Figure 11:**
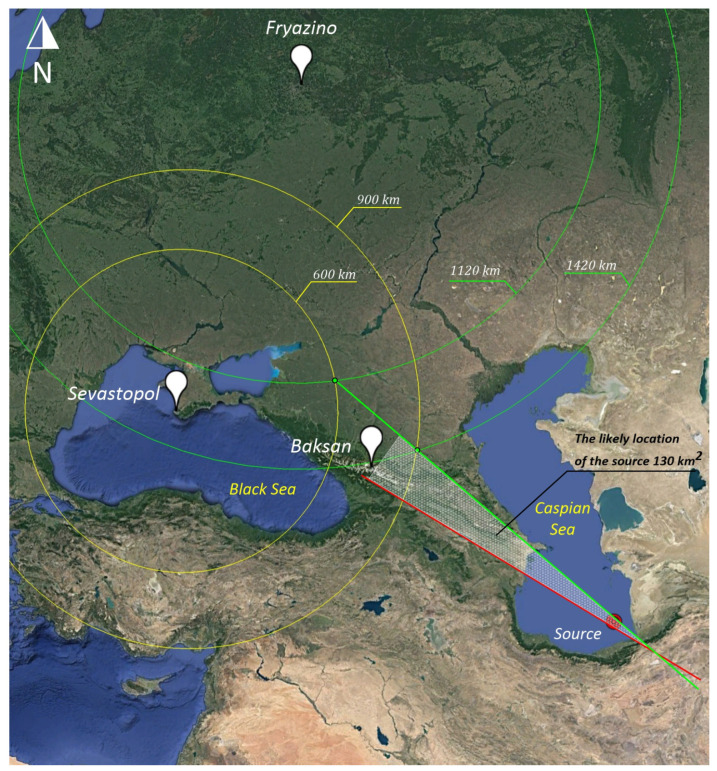
Laser strainmeters “Sevastopol-Fryazino”. Yellow circles of Sevastopol point. Green circles Fryazino point. The red and green lines represent the direction to the source.

We draw two circles with the center of the point Sevastopol with radii of 600 and 900 km, as shown in Figure 11. We calculate the time of the wave arrival from Sevastopol to Fryazino as 734 − 570 = 164 s. We calculate the distance, taking into account the time of propagation from Sevastopol to Fryazino (164 × 3.174 = 520 km). We draw two circles with the center of the point Sevastopol with radii of 600 + 520 = 1120 km and 900 + 520 = 1420 km. By the intersections of the circles, we draw two directions to the true source (green lines). Taking into account the fact that two direction lines intersected, we consider the remaining lines invalid. As a result of the intersection, we obtain an area in the form of a triangle with the base in Baksan and the vertex in the point of the line intersection (gray shading). This triangle is the area in which the true source may be located; the square of the triangle is 130 km^2^.

We include the fourth receiver in Solikamsk.

### 3.4. Fryazino–Solikamsk

The distance from the source to Solikamsk is 2460 km. The travel time of the wave from the source to Solikamsk is 2460/3.174 = 775 s. The time of arrival of the wave from Fryazino to Solikamsk is 775 − 734 = 41 s.

**Figure 12:**
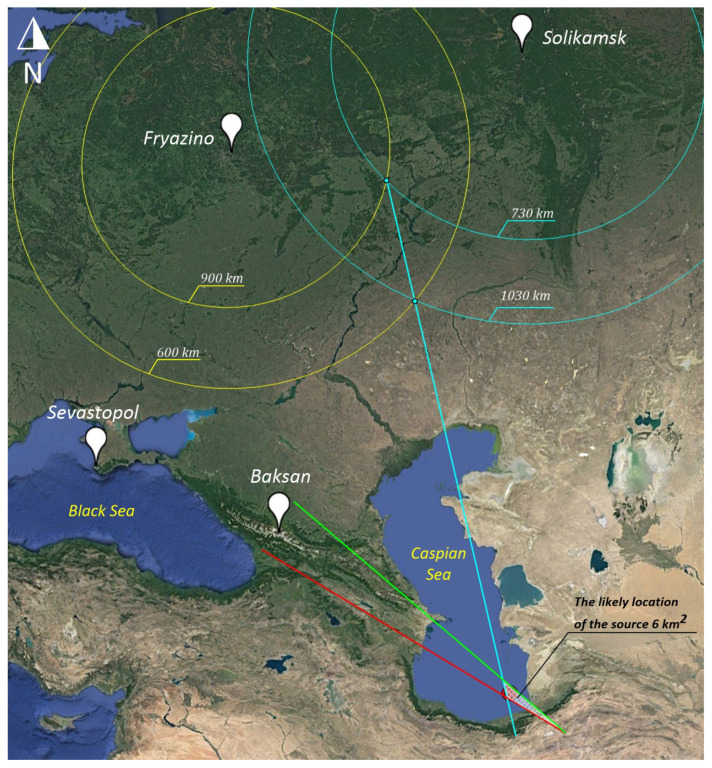
Laser strainmeters “Fryazino-Solikamsk”. Yellow circles of Fryazino point. Blue circles Solikamsk point. The red, green, and blue lines represent the direction to the source.

We draw two circles with the center of the Fryazino point with radii of 600 and 800 km, as shown in Figure 12. We calculate the distance, taking into account the time of propagation from Fryazino to Solikamsk (41 × 3.174 = 130 km). We draw two circles with the center of the point Sevastopol with radii of 600 + 130 = 730 km and 900 + 130 = 1030 km. By the intersections of the circles, we draw the direction to the true source (white line). As a result, we obtain a triangle with a square of 6 km^2^ in which the true source should be located.

Thus, when the fourth strainmeter is included in the system of three, the accuracy of source determination increases 130/6 = 21.6 times.

## 4. Two-Dimensional Laser Strainmeter

At each location of laser strainmeters in the future, it is necessary to create two-coordinate laser strainmeters, the measuring arms of which do not necessarily have to be the same in length, for example [18]. Let us consider the operating principle of this laser strainmeter when determining the possible direction to the source of signals of natural or artificial origin recorded by it. We will assume that the angle between the measuring axes of laser strainmeters is 900. When a longitudinal wave comes at the angle α to the axis of the 52.5 m laser strainmeter, we have the following:

(1) $$\frac{A_{1}}{A_{01}}=\cos\alpha ,\;\frac{L_{12}A_{2}}{A_{01}}=\sin\alpha ,$$

where *A*_1_ is the displacement registered by the 52.5 m laser strainmeter, *A*_2_ is the displacement recorded by the 17.5 m laser strainmeter, and *A*_01_ is the amplitude of the passing wave, *L*_12_ = 2.8. Although the arm length ratio is 3.0, we set it to 2.8, which was determined experimentally when the same pressure load was applied to both strainmeters simultaneously. The coefficient is 2.8, not 3.0, due to the fact that the abutments of these laser strainmeters are located on rocks with different Lamé coefficients, which affect the parameters of the longitudinal wave (velocity, wavelength). The speed and wavelength differ in different rocks. Lamé parameters (also called the Lamé coefficients, Lamé constants, or Lamé moduli) are two material-dependent quantities denoted by λ and μ that arise in strain–stress relationships. Next, we have the following:

(2) $$\alpha =arctg\left(\frac{2.8A_{2}}{A_{1}}\right),$$

Substitute this value α into Equation 1 and we obtain the following:

(3) $$A_{01}=\frac{A_{1}}{\cos(arctg(\frac{2.8A_{2}}{A_{1}}))}$$

When creating two-coordinate laser strainmeters at each measuring point of the planetary seismoacoustic observatory, the task of finding the sources of recorded oscillations/waves is significantly simplified, and the accuracy of determining the coordinates of these sources also increases.

## 5. Conclusions

The reader may have a question about the need to create such a planetary antenna. First of all, the creation of such an antenna is necessary to establish the sources of the recorded signals, which mainly belong to the infrasound range and are of natural and artificial origin. The first experiments in such registration of infrasound disturbances were published in [16], describing signals in the infrasound range recorded by laser strainmeters spaced at a distance of 5217.6 km. When analyzing the processed synchronous experimental data from these two laser strainmeters, short (10–15 wave periods) but very pronounced oscillations are observed, with periods of 6 min 49.6 s and 5 min 41.3 s and correlation coefficients of 0.91 and 0.87, respectively, with some time delay. There is still a question about their origin. There are many such examples, and solving problems of their origin is extremely important for fundamental science.

The creation of the planetary laser interferometric seismoacoustic observatory, consisting of five stationary single-coordinate laser strainmeters and one two-coordinate laser strainmeter, united into a single measuring network with an accurate time clock TRIMBLE 5700 (installed at each measuring range) and capable of recording displacements on their bases with an accuracy of 10 pm in the frequency range from 0 (conventionally) to 1000 Hz and two auxiliary laser strainmeters, will allow us to determine, at any planetary distance, the primary source of deformation infrasound disturbances with primary amplitudes from 100 nm.

The use of this observatory is aimed at solving problems of emergence, development, and transformation of the Earth’s catastrophic processes and phenomena of the lithosphere, hydrosphere, and atmosphere origin, with the possibility of their short-term forecasting. In addition, some fundamental problems of distinguishing between terrestrial and extraterrestrial infrasound disturbances will be solved, which is extremely necessary for an objective interpretation of the results obtained on other planetary installations, for example, those described in [17].

Further, equipping the observatory with other highly sensitive instruments that measure variations in atmospheric and hydrosphere pressure with high accuracy will make it possible to study the influence of geospheric disturbances on each other with some coincidence of their periods of oscillations in the linear and nonlinear cases, which primarily applies to the Earth’s eigen oscillations, eigen oscillations of atmosphere, and eigen oscillations of the World Ocean.

The creation of two-coordinate laser strainmeters at each observation point will significantly improve the accuracy of direction finding of deformation disturbance sources, even by two spatially separated two-coordinate laser strainmeters. And, the introduction of a vertical measuring arm (three-coordinate laser strainmeter) will allow us to classify passing waves into longitudinal, transverse, and Rayleigh waves.

## Figures and Tables

**Table 1:** The data of all laser strainmeters.

№	Strainmeter Location	Coordinates		Length, m	Angle Relative to the “North-South” Line, °
1	Shultz Cape	42°34′48.00″ N	131°09′24.00″ E	52.5	18
2	Shultz Cape	42°34′48.00″ N	131°09′24.00″ E	17.5	110
3	Krasnokamensk	50°03′14.00″ N	118°10′31.00″ E	50	30
4	Svobodny Cape	46°50′54.00″ N	143°25′57.00″ E	20	9
5	Baksan	43°16′37.00″ N	42°41′35.00″ E	75	150
6	Popova Island	42°28′45.00″ N	131°43′30.00″ E	20	46
7	Katsiveli	44°23′24.00″ N	33°58′48.00″ E	30	29

**Table 2:** Distances and time of propagation of oscillations from the source to the receiving laser systems.

№	Strainmeter Location	Distance from Source, km	Propagation Time (t), s	Disturbance Arrival Time (Δt), s	Distance (S), km
1	Svobodny Cape	1570	494	0	0
2	Shultz Cape	2570	809	315	1000
3	Krasnokamensk	2870	904	409	1300

## Data Availability

Third-party data were used. Restrictions apply to the availability of these data. All information about these data and the possibility of obtaining them can be found at chupin@poi.dvo.ru.
